# Evolutionary analysis reveals regulatory and functional landscape of coding and non-coding RNA editing

**DOI:** 10.1371/journal.pgen.1006563

**Published:** 2017-02-06

**Authors:** Rui Zhang, Patricia Deng, Dionna Jacobson, Jin Billy Li

**Affiliations:** 1 Department of Genetics, Stanford University, Stanford, California, United States of America; 2 State Key Laboratory of Biocontrol, School of Life Sciences, Sun Yat-sen University, Guangzhou, P. R. China; University of Michigan, UNITED STATES

## Abstract

Adenosine-to-inosine RNA editing diversifies the transcriptome and promotes functional diversity, particularly in the brain. A plethora of editing sites has been recently identified; however, how they are selected and regulated and which are functionally important are largely unknown. Here we show the *cis*-regulation and stepwise selection of RNA editing during *Drosophila* evolution and pinpoint a large number of functional editing sites. We found that the establishment of editing and variation in editing levels across *Drosophila* species are largely explained and predicted by *cis*-regulatory elements. Furthermore, editing events that arose early in the species tree tend to be more highly edited in clusters and enriched in slowly-evolved neuronal genes, thus suggesting that the main role of RNA editing is for fine-tuning neurological functions. While nonsynonymous editing events have been long recognized as playing a functional role, in addition to nonsynonymous editing sites, a large fraction of 3’UTR editing sites is evolutionarily constrained, highly edited, and thus likely functional. We find that these 3’UTR editing events can alter mRNA stability and affect miRNA binding and thus highlight the functional roles of noncoding RNA editing. Our work, through evolutionary analyses of RNA editing in *Drosophila*, uncovers novel insights of RNA editing regulation as well as its functions in both coding and non-coding regions.

## Author summary

Many important modifications are made to RNA to fine-tune genomic information. One type, Adenosine-to-Inosine (A-to-I) RNA editing, changes certain adenosines to inosines and is essential for the neurological well-being of many animals. Although RNA editing occurs at thousands of sites across the genomes of various animals, the functions of nearly all editing events–particularly those in non-coding regions–have not been studied, and what determines whether particular adenosines across the genome are edited has not been fully explored. Here, using the *Drosophila* genus as model organisms, we analyze the evolution of A-to-I RNA editing to identify a large fraction of both coding and non-coding editing events that are under evolutionary constraint and therefore likely functionally important. We find that non-coding editing events in the 3’UTRs of genes could affect miRNA binding and are associated with a decrease in gene expression levels.

## Introduction

Adenosine-to-inosine (A-to-I) RNA editing converts adenosine to inosine in RNA, which is then read by the cellular machinery as guanosine (G) [1–3]. This process is co-transcriptionally catalysed by adenosine deaminase acting on RNA (ADAR), which recognizes double-stranded RNA (dsRNA) structures as editing substrates [4]. A-to-I RNA editing plays a critical role in neuronal function and integrity through the fine-tuning of editing [5–8]. In humans, changes in editing are associated with neurological disorders such as amyotrophic lateral sclerosis [9] and autism [10]. In *Drosophila*, the knockout of ADAR leads to severe neurological defects such as locomotion impairment, heat sensitive-paralysis, and age-dependent tremors [11].

The widespread presence of A-to-I editing at thousands to millions of sites in various organisms, from flies to humans, has been recently revealed (e.g. [4,12–18]). However, only a handful of sites have been functionally studied [6] and it is unknown what fraction of editing events is functionally important. Recent studies have found that, although human RNA editing events are generally non-adaptive [19] possibly because the vast majority are in primate-specific Alu repeats, high-level nonsynonymous editing events, which cause amino acid changes, are most likely beneficial in humans [20,21]. In species such as squid and *Drosophila*, a significant fraction of editing events are located in coding regions, and many are likely to be beneficial [22–24].

While these studies have highlighted the potential functions of coding editing events, little is known about noncoding events. Few instances of noncoding RNA editing functions have been found: For example, RNA editing in the intron of mammalian ADAR2 alters splicing and thus truncates the protein [25]. In this study, we systematically examine both coding and noncoding editing events by comparing different species in the *Drosophila* genus and find that a surprisingly large fraction of both types are under selective pressure and therefore likely functionally important. Furthermore, we examine the potential functions of editing events in 3’ UTRs using a fly with catalytically inactive ADAR.

Although there are thousands of editing events in *Drosophila*, this only represents a small fraction of the adenosines in the genome, and why particular adenosines are edited is not fully explored. Our analysis of the evolution of RNA editing gives us an opportunity to study how the evolution of RNA editing substrates alters editing. By making incremental changes to editing substrates, evolution has conducted a natural experiment allowing us to test the effects of various changes on RNA editing. Therefore, we can examine the characteristics of editing substrates. dsRNA is a well-known requirement for A-to-I RNA editing [26–28]; we recently found that genetic variants associated with editing level changes are enriched in areas with secondary structure, and the structures around editing sites were more stable in the allele with higher editing levels [29,30]. Furthermore, an RNA sequence motif associated with editing has been found [31]. However, it is unknown how these two factors, dsRNA structure and RNA sequence, work together to determine editing levels. Here, we explore the combined contributions of dsRNA structure and sequence to the evolution of RNA editing in the *Drosophila* genus.

## Results

### Identification and profiling of A-to-I RNA editing across the *Drosophila* genus

**Fig 1:**
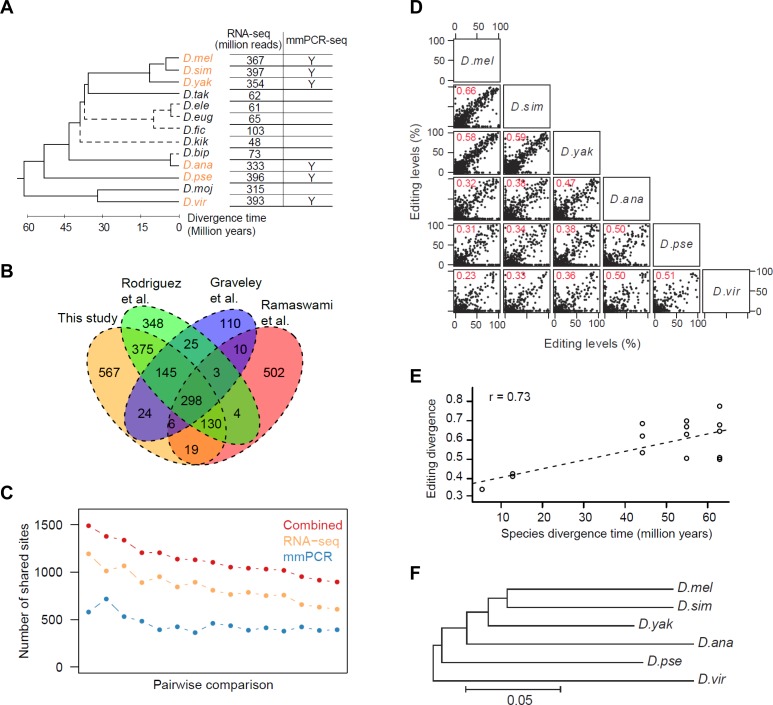
Identification of editing sites and evolution of editing levels. **(A)** A tree representing the schematic phylogeny of *Drosophila* species [43]. All 13 species were used for RNA editing site identification; the 6 species used for editing level evolution analyses are highlighted in orange. *D*. *melanogaster*, *D*.*mel; D*. *simulans*, *D*.*sim; D*. *yakuba*, *D*.*yak; D*. *eugracilis*, *D*.*eug; D*. *takahashii*, *D*.*tak; D*. *ficusphila*, *D*.*fic; D*. *elegans*, *D*.*ele; D*. *kikkawai*, *D*.*kik; D*. *bipectinata*, *D*.*bip; D*. *ananassae*, *D*.*ana; D*. *pseudoobscura*, *D*.*pse; D*. *mojavensis*, *D*.*moj; D*. *virilis*, *D*.*vir*. The divergence time between *D*.*ele*, *D*.*eug*, *D*.*fic*, *D*.*kik*, *D*.*bip* and others are unknown; the dotted lines in the tree only represent the topology. The data used for each species are indicated. (**B**) Venn diagram showing sites reported in this study and three previously published studies. (**C**) Numbers of sites used for pairwise comparisons between the 6 selected species derived from the RNA-seq, mmPCR-seq and combined sets. Sites covered by ≥50 reads were included. The average numbers of the male and female whole body data are shown. (**D**) Pairwise comparison of editing levels (male whole body data), with the Spearman’s rho values in red. Sites covered by ≥50 reads were included. A site that was not an ‘A’ at the DNA level was defined as having an editing level of 0%. (**E**) Editing level divergence distance values (1 –Spearman’s rho) plotted against estimated divergence time for all possible species pairs. The dashed line indicates the linear regression. (**F**) Neighbor-joining tree with branch lengths inferred using editing level (male whole body data) distance (1- Spearman’s rho) for all pairs of species. Only sites edited in both species were used for analysis.

We first assembled a master list of editing sites in the *Drosophila* genus to use for our analyses. Although we and others have recently identified a large number of RNA editing sites in *D*. *melanogaster* (*D*.*mel*) [4,13,16], it is unlikely that their discovery has been saturated. To discover more editing sites in *D*.*mel* and identify additional editing sites unique in other *Drosophila* species, we applied our previously developed methods [16] to RNA-seq data (male and female whole body) from 13 *Drosophila* species (**S1 Table**). We identified 627 novel exonic editing sites, including 545 novel ones edited in non-*D*.*mel* species only (**Fig 1A and 1B, S1A and S1B Fig, S2 Table**). We combined this list with three other sets of sites identified in *D*.*mel* [4,13,16]. Using RNA-seq data from a *D*.*mel* ADAR null mutant [16], we estimated that the false positive rates of these four sets range from 1 to 7% (**S1C Fig**). We removed the false positive sites to obtain 2,380 exonic editing sites (counting orthologous sites only once) (**S2 Table**). These sites are located in 909 genes, preferentially in coding regions and 3’UTRs. 56% alter amino acid coding (**S1D and S1E Fig**).

We next used publicly available RNA-seq data, supplemented with our targeted sequencing assay covering ~600 editing sites, to measure the editing levels of the 2,380 exonic editing sites in *D*.*mel* and 5 other *Drosophila* species that spanned a range of evolutionary distances from *D*.*mel* and had well assembled genomes and deeper RNA-seq coverage (**Fig 1A**). To accurately measure editing levels in the RNA-seq data, only sites covered by ≥20 or 50 reads (medium or high stringency) were included, which leads to an average of 95 or 127 reads per editing site, respectively. While RNA-seq has been used to quantify RNA editing [12,16], its inaccuracy in lowly and moderately expressed genes hinders the accurate measurement of a consistent set of sites. Therefore, to validate and extend measurements in the RNA-seq data, we used the microfluidic multiplex PCR and deep sequencing (mmPCR-seq) assay [32] to quantify the editing levels of an average of ~600 sites in male and female whole body samples of the 6 selected species (**S3 Table, S4 Table**). We obtained ~2,400 reads per editing site per sample (**S2A and S2B Fig**). Editing levels are highly consistent between biological replicates (**S2C Fig**) and with the RNA-seq quantification when sufficient RNA-seq reads are available (**S2D Fig**). Finally, we combined the RNA-seq and mmPCR-seq datasets to obtain a more comprehensive and accurate RNA editing level measurement (**Fig 1C**).

### The contribution of RNA primary sequence and secondary structure to editing divergence

When we compared editing levels between all pairs of the six species (**Fig 1D, S2E Fig**) and, separately, eight *D*.*mel* strains (**S2F Fig**), we found that there was a clear positive correlation between editing level divergence and species divergence time (**Fig 1E**). Furthermore, the editing level divergence tree recapitulates the topology of the phylogenetic tree (**Fig 1F**). These observations suggest that editing level evolution is generally neutral.

Since ADAR expression levels were very similar across species (**S3 Fig)**, we reasoned that changes in *cis* regulatory elements may account for editing level variation at individual sites. *Cis* regulatory elements, namely the primary ADAR sequence motif and dsRNA structure around editing sites, are believed to play an important role in editing regulation across species, as demonstrated in a few case studies [23,28,33–35] and our recent larger scale studies [29,30]. Therefore, we first examined whether the underlying mechanism behind RNA editing divergence between pairs of species involved both RNA sequence motif and dsRNA structure. We also distinguished between sequence differences that removed editing entirely and ones that merely tweaked editing levels.

**Fig 2:**
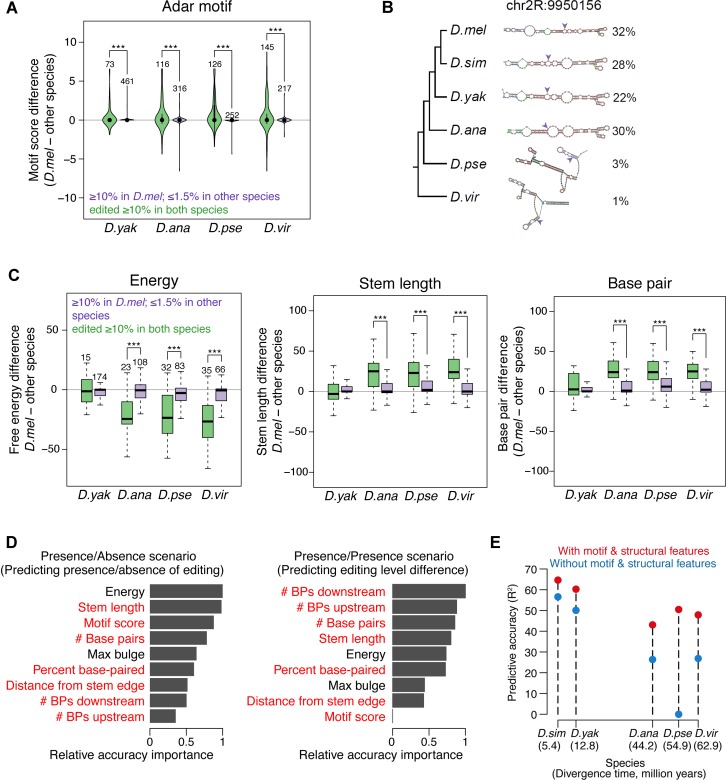
*Cis* regulatory features explain and predict the divergence of RNA editing levels. (**A**) The difference in ADAR binding motif scores between sites that are edited (≥10%) in *D*.*mel* but not edited (≤1.5%) in other species (green) compared to the difference in sites that are edited (≥10%) in both species (purple). *: *p* ≤ 0.05, **: *p* ≤ 0.01, ***: *p* ≤ 0.001 (one-tailed Mann-Whitney U test) (**B**) An example of the relationship between structure and editing level changes across species. The editing site is indicated by the purple arrows. (**C**) The difference in free energy, stem length, and paired bases between sites that are edited (≥10%) in *D*.*mel* but not edited (≤1.5%) in other species and control sites that are edited (≥10%) in both species. The numbers of editing sites are indicated above the bars. *: *p* ≤ 0.05, **: *p* ≤ 0.01, ***: *p* ≤ 0.001 (one-tailed Mann-Whitney U test) (**D**) Relative importance of ADAR motif and dsRNA structural features to predict the establishment of editing (left) and the variation in editing levels (right). In the Presence/Absence editing scenario (left), given that a site was edited (≥10%) in one species, we predicted whether sites were edited (≥10%) or not edited (≤1.5%) in another species. In the Presence/Presence editing scenario (right), given the editing level in one species, we predicted the editing level in another species, requiring that the sites were edited (≥10%) in both species. The *D*.*mel*—*D*.*vir* comparison is shown. Features with positive and negative relationships with presence of editing (left) or increase of editing level (right) are marked in red and black, respectively. (**E**) Comparison of predictive accuracy of editing levels of two models (with or without motif and structural features). Given the editing levels in *D*.*mel*, editing levels in each of the other species were predicted using the random forests model. Sites with editing level ≥10% in at least one of the paired species were included in the analysis.

ADAR’s preferred sequence motif, in particular the triplet containing nucleotides immediately adjacent to the edited adenosine [31], is essentially identical in our six selected species (**S4 Fig**). To evaluate the effect of ADAR motif changes on editing level variation, we first deduced the ADAR motif weight matrix and used it to calculate motif scores for each of the 16 possible nucleotide triplets (**S5 and S6 Tables)**. We compared “Presence/Absence” sites, whose editing was present (≥10% level) in the “anchor” species but absent (≤1.5% level) in the other species under comparison to the control “Presence/Presence” sites, which were edited (≥10%) in both species. With *D*.*mel* as an example anchor species, we found that ~25% of Presence/Absence sites had higher motif scores in *D*.*mel*, while only 8% of them had higher motif scores in the other, non-edited species, indicating an excess of better ADAR motifs in the “anchor” species (*p* = 0.065, 1.9e-3, 5.7e-5, and 6.4e-3 for *D*.*mel* vs. *D*. *yak*, *D*. *ana*, *D*. *pse*, and *D*. *vir*, binomial test) (**Fig 2A**). Strikingly, <1% of the “Presence/Presence” control sites had motif differences between species, although many of the sites were edited at different levels between two species. We observed similar results when performing the same analyses using the other 5 species as the anchor (**S5A Fig**). Therefore, ADAR motif preference plays an important role to determine whether a site is edited rather than how much a site is edited.

To examine the effect of secondary structure on editing level variation, we used a computational pipeline we previously developed [29] to predict editing complementary sequences (ECSs), the sequences base-paired with editing sites, for 1,664 (70%) editing sites in at least one species. Using these ECS predictions, we found that editing differences generally correlate well with secondary structure changes across species as exemplified in **Fig 2B**. This observation is also supported by our recent analyses of secondary structure changes within *D*.*mel* strains [29].

To systematically evaluate how secondary structure changes contribute to editing variation across *Drosophila* species, we dissected the dsRNA structures into 8 structural features and examined their roles separately (**Materials and Methods**). Using the set of Presence/Absence editing sites and its control set of Presence/Presence sites described above, we observed that Presence/Absence of RNA editing best correlated with free energy, stem length, and number of paired bases (**Fig 2C**, **S5B Fig**). This suggests that the stability and length of the RNA duplex play an important role for editing.

While we showed that both motif and dsRNA structure changes are associated with editing changes, their relative importance is unknown. To study how these two regulatory elements work together, we determined the relative importance of the features for the establishment of editing in Presence/Absence sites and the variation in editing levels observed in Presence/Presence sites. We used random forests, a machine learning technique [36], to predict the changes of editing between species using the motif and structural feature changes as predictor variables (**Materials and Methods**). The difference in the prediction accuracy before and after permuting the predictor variable is used as an importance measure (**Fig 2D**). For both scenarios, structural features such as folding energy, base-pairing, and stem length were important. While motif score and maximum bulge size were important in determining the presence or absence of editing, they were less informative for explaining editing level variation. Thus, our observations suggest that both motif and structural features determine whether ADAR can bind and edit a substrate, while changes in structural features contribute to editing level variation. It was previously shown that mutations in the ADAR motif can increase or decrease editing levels *in vitro* [26]. But our data suggests that structural features are generally used to tune editing levels *in vivo*, perhaps because such tuning allows for greater flexibility.

To estimate the contribution of *cis-*regulatory elements to editing divergence, we examined how well these features could be combined together to predict changes in editing using random forests [36]. The difference in the predictive accuracy between two models, with and without ADAR motif and dsRNA structure features, is used as a measure of the contribution of *cis-*elements to editing divergence. In particular, given the editing levels in *D*.*mel*, we predicted the editing levels of orthologous sites in other species. The predictive accuracy of the model with motif and structural features are substantially higher than those of the model without these features (**Fig 2E**). Therefore, a large portion of the editing level divergence across species can be accounted for by changes in *cis* regulation. Taken together, our *cis* regulatory element analyses reveal the structural landscape of editing and demonstrate how *cis* regulatory element changes lead to editing changes.

### The birth and persistence of RNA editing during evolution

**Fig 3:**
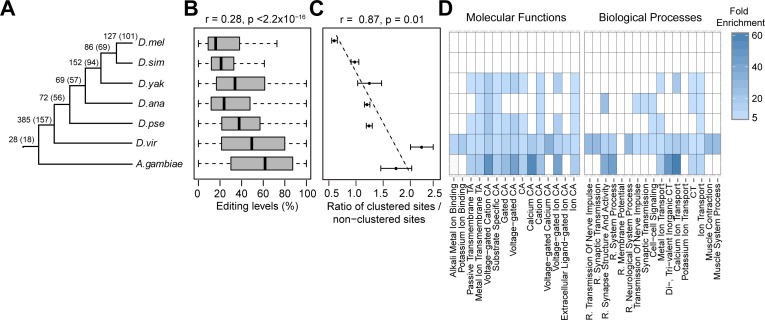
Characterization of the evolution and function of edited genes. (**A-D**) The relationship between age of editing sites and editing levels, the degree of clustering, and gene function. (**A**) Depiction of the species and age groups analyzed; numbers above the branches denote the numbers of *D*.*mel* editing sites in each age group and numbers in parentheses denote the numbers of genes in each age group. (**B**) The relationship between age of editing sites and their representative editing levels. Spearman's ρ is indicated. (**C**) The relationship between age of editing sites and the degree of clustering. We defined a site as clustered if the neighboring site is within a certain distance (30, 40, and 50bp). Error bars, standard deviation. (**D**) The relationship between age of editing sites and function of edited genes. CT: Cation Transport; R.: Regulation of; CA: Channel Activity; TA: Transporter Activity.

Our cross-species data allowed us to examine how RNA editing events may be born or persist during evolution. We used a combination of phylostratigraphy and transcriptome data to deduce the evolutionary age of each *D*.*mel* editing site (**Fig 3A, Materials and Methods**). We found that evolutionarily long-lived (older) editing sites have higher editing levels than younger sites (**Fig 3B**), suggesting that the older sites with higher editing levels might be optimized for ADAR binding and editing. We observed similar results when using the other *Drosophila* species as the anchor (**S6 Fig**). Our observation that older sites are much more likely to be clustered with other nearby editing sites (**Fig 3C**) further supports the idea that the sequences around older editing events can be edited at multiple positions and thus might be optimized for editing. Additionally, we found that the correlation of editing levels between species for old editing sites is higher than that for young editing sites (**S7 Fig**), suggesting that older sites are under stronger purifying selection. Furthermore, we examined the functional enrichment of genes containing the younger and older editing sites. We observed no significant enrichment for genes containing the younger editing sites. However, the older sites gradually became enriched in genes with functions that are mostly neuron-related (**Fig 3D)**. This data agrees with the mostly neurological and behavioral phenotypes of ADAR knockout flies [11], and suggests that older editing sites may have neurological functions.

### RNA editing is selected to occur in slowly evolved genes

**Fig 4:**
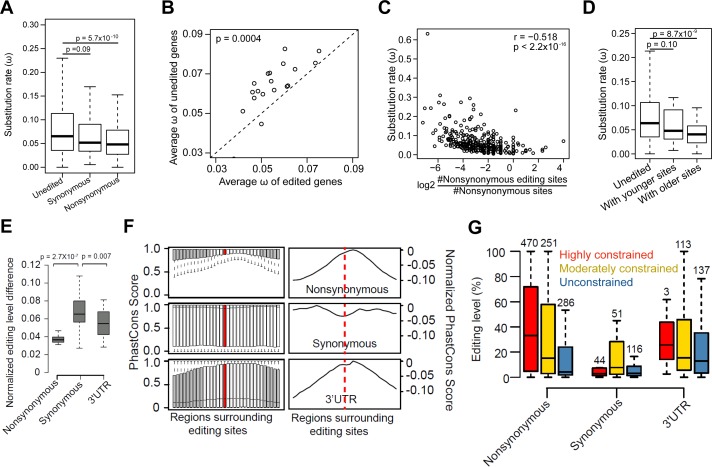
Divergence of editing levels and DNA sequences surrounding nonsynonymous, synonymous, and 3’UTR editing sites. (**A**) The comparison of Omega (ω) between genes with nonsynonymous editing sites, genes with synonymous editing sites only, and unedited genes. Unedited genes are genes without any exonic editing sites. *P*-values were calculated using the Mann-Whitney U test. (**B**) The comparison of average ω between edited and unedited genes for various Gene Ontology (GO) terms. GO terms with at least 20 edited genes were used for the analysis. *P*-value was calculated using the paired-sample Wilcoxon test. (**C**) The relationship between ω of edited genes and the normalized number of nonsynonymous editing sites per gene. The number of nonsynonymous editing sites is normalized by the number of nonsynonymous sites between *D*.*mel* and *D*.*sim* in the gene. Spearman's ρ is indicated. (**D**) The comparison of ω for genes with younger or older *D*.*mel* editing sites. “Genes with younger sites” refer to genes with editing sites conserved in *D*.*mel* or *D*.*sim* only. “Genes with older sites” refer to genes with editing sites conserved beyond *D*.*sim*. *P*-values were calculated using Mann-Whitney U test by comparing these genes with unedited genes. (**E**) The pairwise comparisons of normalized editing level differences between *D*.*mel* strains for nonsynonymous, synonymous, and 3’UTR sites. Sites edited at least 20% in one or both strains in the pairwise comparisons were used. For each editing site, the normalized difference is the ratio of editing difference to mean editing level. *P*-value was calculated using Paired-sample Wilcoxon test. The un-normalized editing level difference is shown in **S9A Fig**. (**F**) Conservation of DNA sequences surrounding *D*.*mel* editing sites. Left: the PhastCons score distributions for the 60 bp regions flanking the editing sites are plotted separately for nonsynonymous, synonymous and 3’UTR sites using a 30 bp window size (**Materials and Methods**). Regions that are significantly different (Kolmogorov-Smirnov Tests, fdr corrected *p* value ≤ 0.01 and D (Kolmogorov–Smirnov statistic, i.e. the distance between two conservation score distributions) ≥ 0.05) from the editing loci (centered, colored red) are colored gray. Right: normalized conservation score, i.e. minus D statistic of the flanking regions of editing loci relative to the region with the highest conservation score. The dashed line indicates the editing site. (**G**) The comparison of editing levels between highly, moderately constrained and unconstrained *D*.*mel* sites. For this analysis, we used the representative editing level of each editing site, which is the maximum editing level across 59 *D*.*mel* RNA-seq datasets, including 30 developmental stages and 29 different tissues (**Materials and Methods**). The editing levels of nonsynonymous and 3’UTR sites were significantly higher than that of synonymous sites (*p* < 8.7Х10^−12^ and *p* < 6.2Х10^−11^, Mann-Whitney U Test). The editing levels of constrained sites were significantly higher than that of unconstrained sites (*p* = 6Х10^−8^). The numbers of editing sites are indicated above the bars.

To determine whether editing tended to affect slowly or quickly evolving genes, we also examined the evolution rate of edited versus unedited genes. We only analyzed RNA editing sites identified from *D*.*mel* RNA-seq data alone [4,13], in order to avoid potential bias from using sites identified from cross-species comparisons. The evolution rate was measured using Omega (*Ka/Ks*), the ratio of the nonsynonymous site substitution rate (*Ka*) to the synonymous site substitution rate (*Ks*) [37]. We found that genes with nonsynonymous editing sites tend to evolve more slowly than unedited genes (**Fig 4A, S8A Fig**). This pattern persists when comparing genes with and without editing sites within the same functional categories (**Fig 4B**). Furthermore, the number of nonsynonymous editing sites per gene is negatively correlated with the evolution rate of edited genes (**Fig 4C, S8B & S8C Fig**). In contrast, no significant correlation was observed for genes with intronic editing sites (**S8D Fig**). Consistent with the findings above, we found that younger sites reside in genes evolving at a rate similar to the rest of the genome, while older editing sites tend to be in slowly evolved genes (**Fig 4D**). Additionally, neuronal genes evolved more slowly (**S8E Fig**). Taken together, these results suggest that individual editing events arise in genes of diverse functions and are subsequently selected to remain in slowly evolved genes, particularly with neuronal functions.

### Both nonsynonymous and 3’UTR editing events are conserved and more likely to be functional

We next separated exonic *D*.*mel* editing sites based on whether they resulted in nonsynonymous, synonymous, or 3’UTR editing (5’UTR sites were not examined because only a few were available) and examined their evolution with respect to both editing levels and surrounding DNA sequences. First, we examined the editing levels of the eight *D*.*mel* strains. The editing levels of nonsynonymous sites are least variable, suggesting the strongest selective pressure. Unexpectedly, the editing levels of the 3’UTR sites are much less variable than those of the synonymous sites, indicating the presence of purifying selection around the 3’UTR sites (**Fig 4E, S9A Fig**). We observed a similar pattern between species, except that the editing levels of 3’UTR sites were more variable, probably because of the increased sequence divergence rate in noncoding regions between species (**S9B and S9C Fig**). Second, we examined the conservation levels of DNA sequences surrounding editing sites. We used RNA editing sites identified from *D*.*mel* RNA-seq data alone [4,13], in order to avoid the potential bias of using sites identified from the cross-species comparisons. We found that, compared to more distal flanking regions, the regions spanning editing sites and separately, their ECS sequences, had a decreased sequence divergence rate for nonsynonymous and 3’UTR sites, but not for synonymous sites (**Fig 4F, S9D Fig**). This is likely due to the evolutionary constraint in maintaining the dsRNA structure for many nonsynonymous and 3’UTR sites. Additionally, we observed higher editing levels of nonsynonymous and 3’UTR sites (**Fig 4G**). These data suggest that a large number of nonsynonymous and 3’UTR, but not synonymous, sites are functionally important.

Our observations prompted us to identify sites that were under evolutionary constraint and thus more likely to be functional (**S2 Table**). By examining the conservation of the regions spanning the editing sites and the more distal flanking regions, we categorized each editing site as highly constrained, moderately constrained, or unconstrained (Materials and Methods). We found 514 highly constrained and 466 moderately constrained sites (**S2 Table**). As expected, the surrounding DNA sequences of evolutionarily older sites tended to be highly or moderately constrained (**S10 Fig**). In addition, the highly constrained nonsynonymous and 3’UTR sites had the highest editing levels, and the moderately constrained sites had higher editing levels than the unconstrained ones (**Fig 4G**), further supporting the functional importance of highly and moderately constrained sites. If one assumes that lowly edited (<1.5%) events are functionally neutral and uses the fraction of lowly edited sites in highly or moderately constrained regions as a baseline, one can roughly estimate that 14.2% (= 75.4%–61.2%, Fisher’s exact test p-value = 5.5e-05) of nonsynonymous editing events and 12.0% (= 50.5%–38.5%, Fisher’s exact test p-value = 0.048) of 3’UTR editing events are in highly or moderately constrained regions and therefore likely functionally important. For synonymous events, the difference is not significant (Fisher’s exact test p-value = 0.42, difference = 2.7% = 45.1%–42.4%). (The fraction of editing events that are functionally important could be greater than this estimate if some lowly edited events are functionally important.) This further supports the idea that a fraction of nonsynonymous and 3’UTR editing events are likely functionally important.

### 3’UTR editing is associated with mRNA expression changes

**Fig 5:**
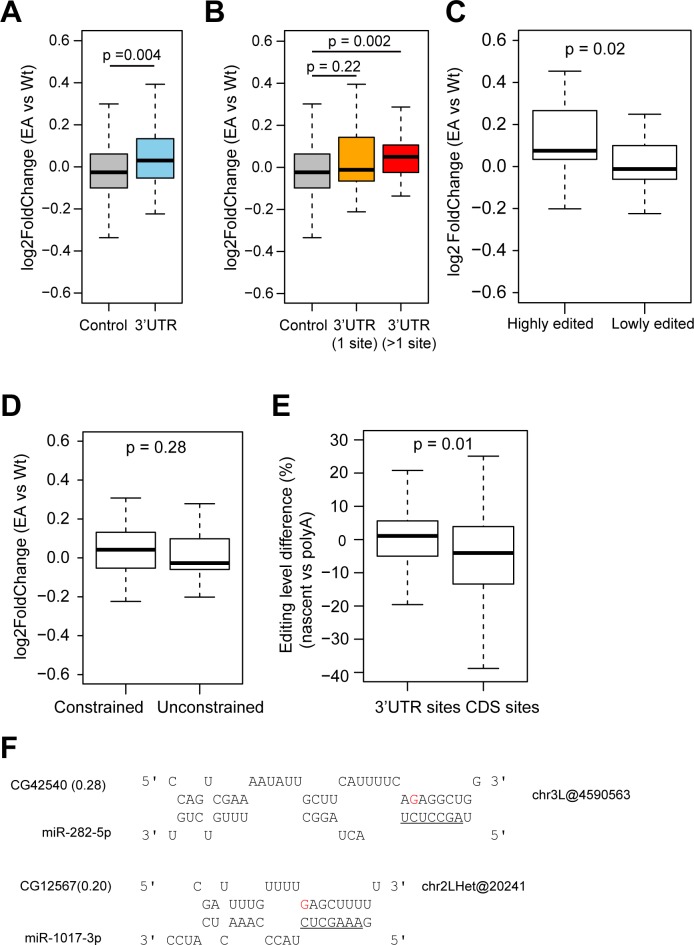
3’UTR RNA editing is associated with gene expression changes. (**A-B**) Gene expression difference between EA mutant and wild type (wt) flies. 3’UTR, genes with 3’UTR editing sites; 3’UTR (1 site), genes with one 3’UTR editing site; 3’UTR (>1 site), genes with more than one 3’UTR editing site; control, genes with 5’UTR or coding editing sites only. Only sites with editing level >5% in the wild type flies were used for analysis. *P*-value was calculated using the Mann-Whitney U Test. (**C**) Gene expression difference between EA and wt flies for genes with highly edited (≥50% editing level) or lowly edited (<50%) 3’UTR sites (**D**) Gene expression difference between EA and wt flies for genes with constrained or unconstrained 3’UTR sites. (**E**) Editing level difference between nascent and polyA RNA-seq for 3’UTR sites and coding sites. *P*-value was calculated using the Mann-Whitney U Test. Only sites with read coverage ≥20 in both datasets and editing level >5% in the nascent RNA-seq data were used for analysis. (**F**) Editing creates putative miRNA target sites. Editing sites are highlighted in red. miRNA seed regions are underlined. Gene expression difference between EA and wt flies is listed in parentheses.

While nonsynonymous amino acid changes affect protein sequence, the function of 3’UTR editing is unclear and under studied. 3’UTR regions often play a regulatory role in gene expression [38]. Therefore, we examined gene expression changes when RNA editing is removed, using RNA-seq data of an ADAR deaminase inactive (EA) mutant fly we generated, which contains a point mutation (E374A) that abolishes the catalytic activity of ADAR but retains the protein [39]. This allows us to examine only the editing-dependent function of ADAR, rather than other possible functions of ADAR. We found that the expression levels of 3’UTR-edited genes increased more in the ADAR mutant than genes edited elsewhere (p = 0.016) (**Fig 5A**). This effect was more dramatic in genes that were edited in more than one site in the 3’UTR (p = 0.004) (**Fig 5B**). Additionally, we found that the expression levels of genes with high 3’UTR editing levels or constrained 3’UTR sites increased more than those of genes with low editing levels or unconstrained sites, respectively (**Fig 5C and 5D**). Thus, 3’UTR editing is associated with changes in gene expression, and this further supports the functional importance of 3’UTR editing.

The association of the presence of 3’UTR editing and decrease in gene expression prompted us to hypothesize that RNA editing in the 3’UTR may lead to mRNA degradation. To test this, we compared editing levels from nascent RNA-seq to those from polyA+ RNA-seq in publicly available data^4^ (**S1 Table**). Because RNA editing is co-transcriptional [4], one would expect the editing levels of nascent RNA to be lower than those of mature polyA+ mRNA. For CDS sites, as expected, RNA editing levels are indeed lower in nascent RNA than polyA+ mRNA (**Fig 5E**). On the contrary, 3’UTR editing sites have very similar editing levels in nascent RNA and polyA+ mRNA (**Fig 5E**). This suggests that, among other possibilities, the edited 3’UTR transcripts may be degraded post-transcriptionally, thus lowering the editing levels in the mature polyA+ mRNA. Further experiments are needed to provide direct evidence for this hypothesis.

We further explored the possible roles of miRNAs in regulating gene expression via differential binding to edited and unedited 3’UTRs [40,41]. We examined if editing sites create or destroy putative miRNA targets for genes with expression differences between WT and catalytically inactive EA flies (log_2_ Fold change >0.2 or <-0.2, 14 genes in total). We only used miRNAs that are co-expressed in fly male head samples for target prediction. We identified two editing events that create binding sites for miR-282-5p and miR-1017-3p; in both cases, the host genes have increased gene expression levels in the EA mutant compared to the WT flies (**Fig 5F**). Therefore, RNA editing in the 3’UTR may affect miRNA binding, thus at least partially explaining the gene expression.

## Discussion

In this work, we examined the birth, death, and persistence of editing events as well as the quantitative changes of editing levels during ~60 million years of evolution in the *Drosophila* genus. We used changes in both RNA primary sequence and secondary structure to predict differences in editing level between species. This highlights the importance of local cis-regulatory changes in the evolution of individual RNA editing events, consistent with the findings of recent studies of RNA editing variation across more closely related *Drosophila* [29,30], and shows that what is known about editing cis-regulation can account for a large fraction of the variation observed. We gained insights into the *cis* regulatory architecture of RNA editing and demonstrated how these features can be used to explain and predict the occurrence of an editing event as well as differences of RNA editing across species. For example, unexpectedly, we found that the ADAR binding motif is a feature that is a lot more important to determine whether a site can be edited compared to what level a site is edited (**Fig 2D**). Our work lays a foundation for future work towards a *cis* regulatory code of RNA editing.

In addition, our data suggests that RNA editing is selected to be enriched in slowly evolved neuronal genes. While neuronal genes evolve slowly, the nervous system acquires high complexity at least partially through the means of RNA editing. Thus, RNA editing, rather than nucleotide substitution at the genomic DNA level, may be the preferred evolutionary means of fine-tuning neuronal functions.

While this manuscript was under preparation, another study on *Drosophila* RNA editing evolution was published [42]. Yu and colleagues examined the evolution of RNA editing in the *Drosophila* genus and found that editing events conserved in at least two members of a gene family were enriched in genes with neurological functions and in regions subject to purifying selection. These conserved editing events tended to cause amino acid changes, which is consistent with the idea that nonsynonymous editing events are more likely functionally important. This is echoed by our analyses that employ a targeted deep sequencing method to achieve much more accurate measurements of RNA editing levels. We both find that highly conserved editing events tend to be nonsynonymous, in neurological genes, and in regions or genes with a lower substitution rate.

In addition to examining coding events, our work delves further into the often ignored non-coding RNA editing events. We found that 3’UTR editing is evolutionarily constrained at the DNA level and highly conserved at the RNA level. This is surprising to us given that the non-coding 3’UTRs are generally not evolutionarily conserved compared to coding regions. While the nonsynonymous editing events may have a more obvious function because of their ability to alter amino acid sequences, our work suggests that many 3’UTR sites are under negative selection. Thus, we identify a large fraction of both nonsynonymous and 3’UTR editing events that are likely functionally important. Our list of candidate functional sites will be valuable for further identification and characterization of truly functional, individual editing sites among the large number of sites that have been recently identified (e.g. [4,12–14]).

Our work further provides evidence of potential functions of 3’UTR editing events. Our finding that 3’UTR editing is associated with mRNA degradation *in vivo* suggests a new role for RNA editing in the regulation of RNA expression levels. We also pinpoint examples of 3’UTR editing sites that alter miRNA binding, thus affecting gene expression. Our findings would add another element to the array of tools by which RNA editing could fine-tune gene function in complex neurological systems. Further experimental work needs to be done to establish whether 3’UTR editing causes gene expression changes, and to explore the mechanism of action. In sum, our analyses provide strong evidence for a set of functional RNA editing sites and indicate an association between 3’UTR editing and mRNA regulation.

By comparative evolutionary analyses, we found that nonsynonymous and 3’UTR sites are edited at high levels and located in highly or moderately constrained genomic regions. These data suggest that nonsynonymous and 3UTR edited sites are functionally important. However, it should be noted that little is known about the dominance of post-editing phenotypes, and editing divergence is only moderately correlated with the editing level (**S11 Fig**), so caution should be exercised when using editing level alone as a proxy for fitness or negative selection. The approaches used in this study can be applied to identify functionally important editing events in other species, such as the primates where RNA editing is a lot more abundant particularly in non-coding regions [17].

## Materials and methods

### RNA-seq data collection

We obtained RNA-seq data for 13 *Drosophila* species from the NCBI Sequence Read Archive (SRA) (http://www.ncbi.nlm.nih.gov/sra) and modENCODE project (http://www.modencode.org/). We obtained the *Anopheles gambiae* whole body RNA-seq data from NCBI SRA. A list of datasets is shown in **S1 Table**. We obtained *D*.*mel* yellow white (yw) strain nascent RNA-seq, ADAR null mutant nascent RNA-seq, yw genomic DNA-seq, ADAR wild type and null mutant RNA-seq from NCBI SRA (GSE37232, GSE42815).

### Mapping of RNA-seq reads and calling RNA variants

We adopted a pipeline that can accurately map RNA-seq reads to the genome [12]. In brief, we used BWA [44] to align individual RNA-seq datasets to a combination of the reference genome and exonic sequences surrounding known splicing junctions from available gene models (obtained from the UCSC genome browser). We chose the length of the splicing junction regions to be slightly shorter than the RNA-seq reads to prevent redundant hits. After mapping, we used SAMtools [45] to extract uniquely mapped reads, merged uniquely mapped reads of individual datasets from the same sample, and detected nucleotide variants between the RNA-seq data and reference genome. We took variant positions in which the mismatch was supported by ≥2 reads and both base and mapping quality scores were at least 20. We used additional filters to remove wrongly assigned mismatches as previously described [12].We inferred the strand information of the sites based on the strand of the genes. Regions with bidirectional transcription (sense and antisense gene pairs) were discarded. ANNOVAR was used to annotate the editing sites [46].

### Cross-species position conversion

The LiftOver tool was used to convert genomic positions between different species. Since LiftOver does not provide strand information between two species (for example, a sense strand in one species may correspond to a reverse stand in another species), we obtained the strand information between different species using pairwise alignment data from UCSC genome browser. For the 6 species without genome alignments available on the UCSC genome browser (*D*.*eug*, *D*.*tak*, *D*.*fic*, *D*.*ele*, *D*.*kik*, and *D*.*bip*), the sequences were aligned using Lastz with the following parameters: H = 2000, Y = 3400, L = 4000, K = 2200, O = 400, and E = 30 [47]. Then, these were chained, netted, and converted to axt files using the tools axtChain (with a minimum chain score of 3000), chainPreNet, chainNet, and netToAxt from UCSC [48].

### False positive rate estimation and validation of *D*.*mel* A-to-I sites using wild-type and ADAR null mutant RNA-seq data

RNA-seq data of the wild-type strain and ADAR null mutant were obtained from two recent studies [4,16]. Sequences were mapped as described above. We examined all identified A-to-G sites that are edited in the wild type strain (defined as having more than two altered reads and editing levels ≥ 1.5%). Sites that do not have altered reads in the ADAR null mutant were considered to be genuine A-to-I RNA editing sites. For sites that have altered reads in both the wild-type and ADAR null mutant, statistically significant editing sites in the wild-type were determined by applying Fisher’s exact test to compare the A-to-G occurrences between the wild-type and ADAR null sample [16]. P values were corrected using the Benjamini-Hochberg method, and a confidence level of 0.05 was used as the cutoff.

### Exonic editing site identification in *Drosophila*

We have recently developed a cross-species transcriptome comparison method that accurately identifies exonic RNA editing events [16]. We modified this method and applied it to the transcriptomes of 13 *Drosophila* species (**S1A Fig**). RNA variants were called separately for each species as described above. Shared RNA variants that are present in any of the species pairs were obtained using various frequency cutoffs. As expected, the majority of the resulting RNA variants are A-to-G mismatches, indicative of A-to-I editing (**S1B Fig**). To achieve a high accuracy in editing site calling without a substantial reduction in sensitivity, we chose a frequency cutoff such that the fraction of variants that were A-to-G was at least 80% [26]. In total, we identified a list of 1,564 exonic A-to-I editing sites (**Fig 1B, S2 Table**).

We next combined this list with three other lists [4,13,16]. We estimated the false positive rates of all studies using RNA-seq data from the *D*.*mel* wild type strain and from the Adar null mutant that eliminates RNA editing (**S1C Fig**). We then removed the sites we found to be false positives to obtain 2,380 exonic editing sites.

We note that we did not include a recently published *D*.*mel* editing site list [15] because all our experiments and data analyses had been done before the publication of the study. In addition, the number of novel sites from that study only accounted for 13% of the sites collected in our current list. Therefore, the exclusion of sites from this recent study is unlikely to affect our conclusions.

### Fly collection

A list of all species and strains used for mmPCR-seq are listed in **S3 Table.** All stocks were grown on standard molasses media (Stanford fly media center). Ten whole bodies were collected from adult flies (eclosion + ~5 days) for each sample. The ADAR E374A mutant (“Adar EA”) contains 2 point mutations: C1725T (synonymous) and A1733C (leading to the E374A mutation), was produced using CRISPR reagents described in [49], and is on a *y sc* background, backcrossed to white Canton-S (CS) for 7 generations. For the RNAseq, stocks were grown at 18°C, and heads were collected from 3 day old male adult flies.

### RNA and cDNA preparation

Total RNA was extracted with the RNeasy Kit (Qiagen). After DNase I treatment, 3 ug of total RNA was used to synthesize the cDNA using iScript™ Advanced cDNA Synthesis Kit (Bio-Rad). cDNA was purified with MinElute PCR Purification Kit (Qiagen).

For the ADAR EA mutant RNAseq, total RNA was extracted using RNAdvance magnetic beads (Agencourt), treated using TURBO DNase (Thermo Fisher Scientific), depleted of ribosomal RNA [50], and treated again using TURBO DNase.

### mmPCR-seq

We used a microfluidic multiplex PCR and sequencing method [32] to quantify the RNA editing levels of selected sites. We selected sites that are edited at ≥5% editing levels in adult *D*.*mel* using RNA-seq data for primer design. Using the *D*.*mel* genome, we designed 48 pools of 12 to 15-plex multiplex PCR primers [32], which covered a total of 605 loci, allowing us to examine all of these loci on a single chip. The sizes of the amplicons range from 150 to 350 bp. For distantly related species *D*.*ana*, *D*.*pse*, and *D*.*vir*, we also designed additional multiplex primers, which covered 192, 211, and 232 editing loci, respectively, that could not be amplified using the primers designed for *D*.*mel*. All primer sequences are listed in **S4 Table**.

We next loaded cDNAs and primer pools into the 48.48 Access Array IFC (Fluidigm) and performed target amplification as previously described [32]. PCR products of each sample were then subject to 15 cycle barcode PCR and pooled together. All pools were combined at equal volumes and purified via QIAquick PCR purification kit (Qiagen). The library was sequenced using Illumina HiSeq with 101 bp pair-end reads.

We used the FASTX Toolkit (FASTQ/A short-reads pre-processing tools, hannonlab.cshl.edu/fastx_toolkit) to demultiplex the raw reads. We used Tophat [51] to align the pair-end reads to the corresponding genome.

For editing level quantification, sites covered by ≥50 mmPCR-seq reads were used.

### Quantifying editing levels using two rounds of targeted RNA sequencing

We performed two rounds of targeted RNA sequencing. In the first round, we used mmPCR-seq to amplify and sequence all selected editing loci for all samples from six species with the primers designed for *D*.*mel*. For the non-*D*.*mel* species, because of the presence of the mismatches between some of the *D*.*mel* primers and templates, we expected that the amplification efficiency of different loci would be variable; in addition, some loci could not be amplified. As expected, we found a higher variability in coverage of various loci in non-*D*.*mel* data (**S2B Fig**). Since we are only comparing the ratios of two alleles of the same amplicon, we expect that the amplification efficiency difference should not affect the accuracy of editing level measurement. Both the high reproducibility of biological replicates (**S2C Fig)** and the general consistency between RNA-seq and mmPCR-seq (**S2D Fig)** support this notion.

In the mmPCR-seq data, for closely related species *D*.*sim* and *D*.*yak*, we were able to amplify about 80% and 70% of the selected sites, respectively. For distantly related species *D*.*ana*, *D*.*pse* and *D*.*vir*, we were only able to amplify about 56%, 50% and 48% of the loci, respectively. Therefore for these 3 species, we designed additional multiplex primers for ~210 loci that could be amplified in *D*.*mel*, *D*.*sim* and *D*.*yak* but not in *D*.*ana*, *D*.*pse* and *D*.*vir*. We used these primers for the second round of targeted RNA sequencing. We amplified these loci using a regular PCR machine. All samples were then barcoded, pooled and sequenced in one Illumina MiSeq run with 150 bp paired-end reads.

### Editing level divergence analysis

For editing level divergence related analyses, sites covered by ≥20 reads were used unless otherwise specified. To maximize the number of sites used for analysis, we combined the whole body RNA-seq data with the mmPCR-seq data. For mmPCR-seq, sites with ≥50 reads and editing level differences ≤10% between biological replicates were used. For sites with both RNA-seq and mmPCR-seq measurements, we used the mmPCR-seq measurement. Editing level divergence was defined as 1 –Spearman’s rho, where rho was the correlation between editing levels in two species.

### ADAR binding motif analyses

The motif weight matrix was deduced using the nucleotide triplets (the editing site and its immediately adjacent nucleotides) of highly edited sites (≥50%). The motif weight matrices are very similar across species so we used the average motif weight matrix for analysis. Motif scores of each of the 16 possible nucleotide triplets were calculated by FIMO [52] and then scaled to the range of 0–10.

To maximize the number of sites used for “Presence/Absence” site analysis, we combined the male and female whole body data. For an editing site to be considered as absence, we required that the observed A-to-G frequency was less than 1.5% in all datasets with editing level measurements for the site. For an editing site to be considered as presence, we required that the A-to-G frequency was at least 10% in at least one dataset. To estimate if an absent site has evidence of editing, we examined if the editing level of this site was significantly higher than the typical A-to-G sequencing error rate (1%) [32] based on the read coverage of this site using Binomial Test. For example, with *D*.*mel* as an anchor species, we found 492 Presence/Absence sites. 90% of the Presence/Absence sites had statistically significant higher editing levels in *D*.*mel* (*p*<0.05, Fisher’s exact test). And 99% of the absence sites had similar editing level to the typical A-to-G sequencing error rate, indicating no evidence of editing.

### Editing Complementary Sequence (ECS) predictions

We used the proximal and distal ECS prediction pipelines previously described [29]. Briefly, to predict proximal ECSs, we predicted the secondary structure of the region within 200 bp of each editing site using the programs partition, MaxExpect, and ct2dot from the RNAStructure package [53], and identified ECS-like sequences with at least 20 bases paired in the stems and a max bulge of 8. The same method was used to predict proximal ECSs in all 6 *Drosophila* species. To predict distal, intronic ECSs, we predicted conserved ECSs located in intronic regions. We smoothed phastCons scores using a sliding window of 51 bp [34]. We selected regions that were within 2,500 bp of the editing site and at least 20 bases long with a smoothed phastCons score of at least 0.90 (determined using known intronic ECSs). Next, we folded candidate sequences and identified ECSs as with the proximal ECSs. Since the intronic ECS predictions could bias our analyses towards *D*.*mel*, the species in which they were identified, we used only the proximal ECSs for our cross-species evolutionary analyses. Both the intronic and proximal ECSs were used for comparisons within the *D*.*mel* population.

### ECS and dsRNA structure analyses

We used our ECS predictions for two types of analyses to examine how differences in various features of orthologous sites are related to differences in editing level. First, we examined changes of editing in two scenarios. In the “Presence/Absence” scenario, we examined features of sites that were edited (≥10%) in an “anchor” species and unedited (≤1.5%) in another species. As a control, comparisons were also made in the “Presence/Presence” scenario, in which both the anchor and other species were edited (≥10%). Sites with predicted ECSs that passed the filter in the anchor species were used. (The ECS was not required to pass the filter in the other species since it may or may not be present.) Secondly, we predicted the editing level in a second species, given the editing level in the first species. In this analysis, sites with ECS predictions that passed the filter in at least one of the two species were used.

### RNA editing prediction via the random forests model

We dissected the dsRNA structures into 8 structural features (free energy, stem length, max bulge size, percent of the stem that is base-paired, distance between editing site and closest stem edge, number of paired bases in the entire stem, and, separately, number of paired bases in the stem downstream and upstream of the editing site). To determine the free energy of the dsRNA stem, we joined the editing stem with the complementary region stem by a 100 base linker of adenosines and ran the RNA secondary structure prediction program fold from the RNAStructure package.

The relationship between editing changes and the ADAR motif and 8 structural features was modeled using random forests [36] (R package: randomForest; parameters: ntree = 10000, mtry = 3, importance = TRUE). The random forests algorithm is particularly resistant to overfitting and uses the out-of-bag error (for each iteration, calculated using the unused samples (roughly one-third)) to obtain an unbiased estimate of the accuracy rate [54].

To predict whether a site is edited (“Presence/Absence”), classification trees were used. (Classification trees are decision tree models in which, at each branch, decisions based on features (ie: RNA structure or sequence) are made to classify items (ie: sites).) To predict the editing level changes (“Presence/Presence”), regression trees, which are decision tree models that use continuous values, were used. The relative importance of the features (accuracy importance for classification, percent increase in mean squared error for regression) was calculated by randomly permuting each feature, taking the average difference between the accuracy of predictions using the permuted data and the accuracy using the non-permuted data, and then dividing by their standard deviation [36]. The percent variation (*R*^2^) = 1 –(the mean squared error of the editing level prediction / the variance in editing levels).

For predicting the editing level in one species using the editing level in *D*.*mel* without motif and structural features, there was only one feature, so only one was sampled at each split (parameters: ntree = 10000, mtry = 1, importance = TRUE).

### PhastCons analysis of regions surrounding the editing sites to categorize editing sites by level of evolutionary constraint

To calculate the conservation levels of DNA sequences surrounding the editing sites, we used a 30 bp sliding window size because the editing stem regions are usually 30–50 bp long [24]. For nonsynonymous and synonymous sites, we did not use flanking regions if they fell into a non-CDS region. For 3’UTR sites, we did not use flanking regions if they overlapped with any annotated CDS regions.

To categorize each editing site as highly constrained, moderately constrained or unconstrained, we examined the conservation of the region spanning the editing sites (S_region, 15 bp upstream and 30bp downstream of the editing site) and the more distal flanking region (F_region, 46bp before and after the S_region). We first defined sites with high conservations (average PhastCons score > 0.9) for both S_region and F_region as highly constrained sites. For the remaining sites, we defined sites with a statistically significant higher conservation in the S_region compared to the F_region to be moderately constrained sites, and the ones with similar or lower conservation of S_regions as unconstrained sites. The significance was determined using two-sample Kolmogorov-Smirnov test (fdr corrected p value < 0.05).

### Representative editing level of individual editing sites in *D*.*mel*

We obtained a total of 59 *D*.*mel* RNA-seq datasets, including 30 RNA-seq datasets from 30 developmental stages spanning the whole life cycle and 29 tissue-specific RNA-seq datasets in various tissues dissected from various stages (http://www.modencode.org/). For each dataset, we calculate editing levels for editing sites covered by ≥20 reads. For each site, the maximal editing level across all samples was used as the representative editing level of a site.

### Dating the RNA editing events

We defined the age of each *D*.*mel* editing event using a combination of the male and female whole body data. We used 5 fly species (*D*.*sim*, *D*.*yak*, *D*.*ana*, *D*.*pse*, *D*.*vir*) and 1 mosquito species (*Anopheles gambiae*) representing 6 time points compared to *D*.*mel*. We posited that editing events rarely independently originate from different lineages so the age of each *D*.*mel* editing site was based on the most distantly related species in which sites were also edited (parsimony principle).

To determine if a site is edited, we required the editing level to be ≥2% in at least one dataset. With this cutoff, 89% of the editing sites have significantly higher editing levels than the typical A-to-G sequencing error rate (1%) [32] (p < 0.05, Binomial Test). To determine if a site is unedited, we required that the site be covered by ≥20 reads and that the editing level be ≤1.5% in all datasets. With this cutoff, <1% of the editing sites have significantly higher editing levels than the typical A-to-G sequencing error rate (p < 0.05, Binomial Test). A site that was not an A at the DNA level was defined as unedited. Only sites that could be well defined as edited or unedited in at least 5 species were used for analysis.

Gene ontology (GO) term analysis was done using DAVID [55]. P-values were corrected for multiple hypotheses testing using the Bonferroni method. All statistically significant GO terms (p ≤ 0.01) with at least 5 genes were shown for all age groups except the *D*.*vir* age group for which we showed the top 10 GO terms from the total of 33 significant GO terms.

### Substitution rate omega (ω) analyses

The ω values of each gene were obtained from Larracuente and colleagues [56]. In brief, all single-copy orthologs in the melanogaster group were obtained. Paralogs were excluded because of difficulties in computationally verifying the accuracy of phylogenies and of alignments. We further selected genes that have orthologs in all fly species compared for analysis. The orthologous gene information was obtained from flybase. Only the melanogaster group was used because divergence at silent sites is too great (saturated) beyond the melanogaster group, which prevents an accurate estimation of dS and thus would erode the power to accurately estimate both rates of evolution (dS and ω) [56]. PAML (Phylogenetic Analysis by Maximum Likelihood) was used to calculate ω [37].

### RNA-seq and analysis of gene expression of edited genes

RNA sequencing libraries of the Adar EA mutant and wild type control were produced using the KAPA RNA-Seq Kit (Kapa Biosystems). Libraries were sequenced on an Illumina NextSeq 500 Sequencer using paired-end 75-bp cycles. Reads were mapped using TopHat [51] and DESeq2 [57] was used to obtain gene expression levels. Only genes with editing levels above 5% and at least 20 reads at the editing site were used in the analysis.

### RNA-seq analysis of nascent and polyA RNA-seq data

We obtained nascent and polyA RNA-seq data for yw male head samples from a recent study [4]. Reads were mapped using BWA to a combination of the reference genome and exonic sequences surrounding known splicing junctions, as described above. Only sites covered by at least 20 reads were used in the analysis.

### miRNA binding site analysis

We used the TargetScan algorithm [58] to predict miRNA binding sites for the unedited and edited form of 3’UTRs using head expressed *D*.*mel* miRNAs. miRNAs were obtained from miRBase database Release 20. Male head miRNA expression data was from GSM322543. Only miRNAs expressed in male heads (with >0.1% of the total miRNA reads, 62 miRNAs in total) were used for analysis.

## Supporting information

S1 Fig Identification and characterization of RNA editing sites in *Drosophila*. (**A)** The pipeline for exonic editing identification by the cross-species transcriptome comparison method. (**B**) Relationship between the proportion of detected mismatches that are A-to-G and the minimum editing level (i.e., the percentage of reads with the variant nucleotide). The colors indicate the minimum editing levels. Each colored, vertical line represents the RNA variants shared by one pair of species. Dotted line indicates an 80% A-to-G fraction cutoff. (**C**) The false discovery rate (FDR) of exonic RNA editing site identification for the 4 specified studies. The *D*.*mel Adar*^*-/-*^ mutant RNA-seq data were used to estimate FDRs. The numbers of editing sites are indicated above the bars. (**D**) Distribution of exonic sites in various genic regions. CDS sites are categorized by functional consequence into three groups: nonsynonymous, synonymous, and stoploss changes. (**E**) The density of exonic sites in various genic regions.
(PDF)

S2 Fig The quantification and comparison of editing levels across the 6 selected species. (**A**) The read number (gray bar) and mapping rate (red dot) of each sample. (**B**) The coverage of editing sites for each sample. (**C**) Correlations between editing levels of biological or technical replicates. Sites present in all samples for each species were used for this analysis. All samples are biological replicates except the ones indicated with *. (**D**) Comparison of editing levels measured by mmPCR-seq and RNA-seq. Both the RNA-seq and mmPCR-seq data are from ~5 day adult whole body samples. A minimum number of 20 and 50 reads is required for sites measured with RNA-seq and mmPCR-seq data, respectively. (**E**) Pairwise comparison of editing levels between species. The Spearman’s rho values are indicated in red. Female whole body data is shown. (**F**) Comparison of editing levels measured by mmPCR-seq in eight *D*.*mel* strains. Spearman’s rho values are indicated in red.
(PDF)

S3 Fig ADAR expression in the 6 selected *Drosophila* species. The FPKMs (fragments per kilobase of exon per million fragments mapped) of ADAR and tubulin are shown. The error bars indicate the differences between male and female gene expression levels. The triangles indicate the ADAR expression levels normalized by the tubulin expression levels.
(PDF)

S4 Fig The composition of nucleotides adjacent to editing sites in *Drosophila* species. Nucleotide composition in the position immediately upstream (left) and downstream (right) of editing sites in six *Drosophila* species.
(PDF)

S5 Fig The relationship between *cis* regulatory feature changes and the divergence of RNA editing levels. (**A**) The difference in motif score for editing sites that are edited (≥10%) in one species but not edited (≤1.5%) in the other species under comparison (green), compared to the control in which sites are edited (≥10%) in both species (purple). (**B**) The difference in free energy, number of paired bases and stem length between sites that are edited (≥10%) in one species but not edited (≤1.5%) in the other species under comparison (green), compared to the control in which sites are edited (≥10%) in both species (purple). The *D*.*mel-D*.*sim* comparisons are not shown because there are fewer than 10 editing sites with ECS predictions that are edited ≥10% in *D*.*sim* and not edited in *D*.*mel*. *D*. *pse* and *D*. *vir* are not shown because, for every species pair, fewer than 10 editing sites with ECS predictions are edited ≥10% in them and not edited in the other species under comparison. *: *p* ≤ 0.05, **: *p* ≤ 0.01, ***: *p* ≤ 0.001 (one-tailed Mann-Whitney U test).
(PDF)

S6 Fig The relationship between the age of editing sites and editing levels using non-*D*.*mel* species as the “anchor.” Left of each panel: Depiction of the species and age groups analyzed; numbers above the branches denote the numbers of editing sites in each age group. The anchor species is in bold. Right of each panel: Boxplots of the editing levels in the anchor species for the editing sites in each age group. Spearman’s ρ is indicated. (For the plots using *D*.*mel* as the “anchor,” please see **Fig 3**.)
(PDF)

S7 Fig The correlation of editing levels between *D*.*mel* and *D*.*sim* for young and old sites. Young sites: shared only in *D*.*mel* subgroup (*D*.*mel*, *D*.*sim*, and *D*.*yak*); old sites: sites edited in all *Drosophila* species analyzed. Both Pearson and Spearman correlation coefficients are shown.
(PDF)

S8 Fig The evolution of edited genes. (**A**) The comparison of evolution rate between genes with nonsynonymous sites, genes with synonymous sites only, and unedited genes. Since editing preferentially occurs in neural tissues, to avoid potential bias, we used genes expressed in heads as a control. (**B-C**) The relationship between ω of edited genes and the normalized number of nonsynonymous editing sites per gene between *D*.*mel–D*.*yak* (**B**) or *D*.*mel–D*.*ana* (**C**). Spearman's ρ is indicated. (**D**) The relationship between the number of intronic editing sites per gene and omega. The number of intronic editing sites is normalized by the number of adenosines in the intron. (**E**) The comparison of evolution rate between neuronal genes and all fly protein coding genes. We defined genes with neuron-related GO terms to be neuronal genes. P-values were calculated using the Mann-Whitney U test.
(PDF)

S9 Fig The evolution and function of editing sites. (**A**) The pairwise comparisons of editing level differences between *D*.*mel* strains for nonsynonymous, synonymous, and 3’UTR sites. To minimize the effect of different distributions of editing levels in the three categories, sites edited at ≥20% in one or both strains in the pairwise comparisons were used. P-value was calculated using Paired-sample Wilcoxon test. (**B-C**) The pair-wise comparisons of normalized (B) or unnormalized (**C**) average editing level difference across species. Male whole body data was shown. All sites with at least 20 reads are included. Sites edited at ≥20% in one or both strains in the pairwise comparisons were used. P-value was calculated using the Paired-sample Wilcoxon test. (**D**) Conservation of DNA sequences surrounding ECSs. Left: the PhastCons score distributions for the 60 bp regions flanking the corresponding sites in ECSs that are paired with the editing sites. The results were plotted separately for nonsynonymous, synonymous and 3’UTR editing sites using a 30 bp window size. We only analyzed *D*.*mel* proximal ECSs, in order to avoid potential bias of using evolutionarily conserved intronic ECSs. Since ECSs located in different genic regions likely have different evolution rates, for nonsynonymous and synonymous editing sites, we only examined ECSs located in CDS regions; for 3’UTR editing sites, we only examined ECSs located in 3’UTR regions. Regions that are significantly different (Kolmogorov-Smirnov Tests, fdr corrected p ≤ 0.01 and D ≥ 0.05) from the site paired with editing site (centered, colored red) are colored gray. Right: normalized conservation score, i.e. the D statistic of the flanking regions of edited loci relative to the region with the highest conservation score.
(PDF)

S10 Fig The relationship between age of editing sites and evolutionary constraint. Left: Depiction of the species and age groups analyzed as in **Fig 3A**. Right: The distribution of highly (red), moderately constrained (yellow), or unconstrained (blue) sites in each age group.
(PDF)

S11 Fig The relationship between editing conservation and editing level. Editing conservation is defined as 1 − Absolute value (editing level, *D*.*mel*–editing level, the other species) / (editing level, *D*.*mel* + editing level, the other species). Spearman's ρ is indicated.
(PDF)

S1 Table RNA-seq datasets used for analysis. (XLSX)

S2 Table Annotation of fly exonic A-to-I editing sites. (XLSX)

S3 Table Fly strains used for mmPCR-seq. (XLSX)

S4 Table Primers used in multiplex PCR to amplify RNA editing loci. (XLSX)

S5 Table The ADAR motif weight matrix used for analysis. (XLSX)

S6 Table The motif scores for each of the 16 nucleotide triplets. (XLSX)

S7 Table ECS predictions in D.mel and five other species. (XLSX)

S8 Table Summary of the numbers of predicted ECSs in each species. (XLSX)
