# Supplementary figures and images for: Evolutionary analysis reveals regulatory and functional landscape of coding and non-coding RNA editing
Source: PLoS Genet. 2017 Feb 6;13(2):e1006563. doi: 10.1371/journal.pgen.1006563 (PMC5319793; doi:10.1371/journal.pgen.1006563)

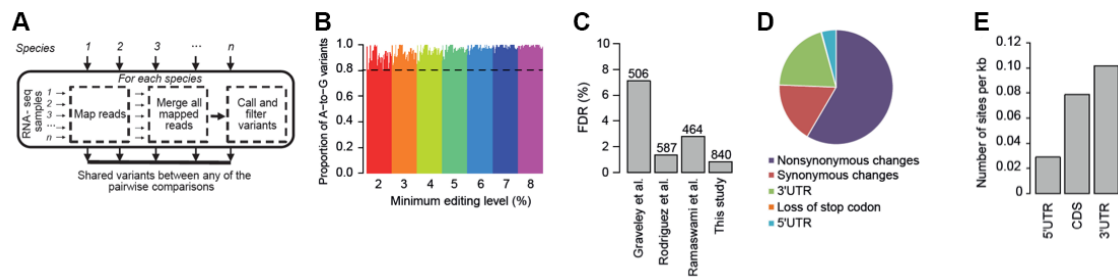

Supplement: S1 Fig — (A) The pipeline for exonic editing identification by the cross-species transcriptome comparison method. (B) Relationship between the proportion of detected mismatches that are A-to-G and the minimum editing level (i.e., the percentage of reads with the variant nucleotide). The colors indicate the minimum editing levels. Each colored, vertical line represents the RNA variants shared by one pair of species. Dotted line indicates an 80% A-to-G fraction cutoff. (C) The false discovery rate (FDR) of exonic RNA editing site identification for the 4 specified studies. The D.mel Adar-/- mutant RNA-seq data were used to estimate FDRs. The numbers of editing sites are indicated above the bars. (D) Distribution of exonic sites in various genic regions. CDS sites are categorized by functional consequence into three groups: nonsynonymous, synonymous, and stoploss changes. (E) The density of exonic sites in various genic regions. (PDF) [file pgen.1006563.s001.pdf]

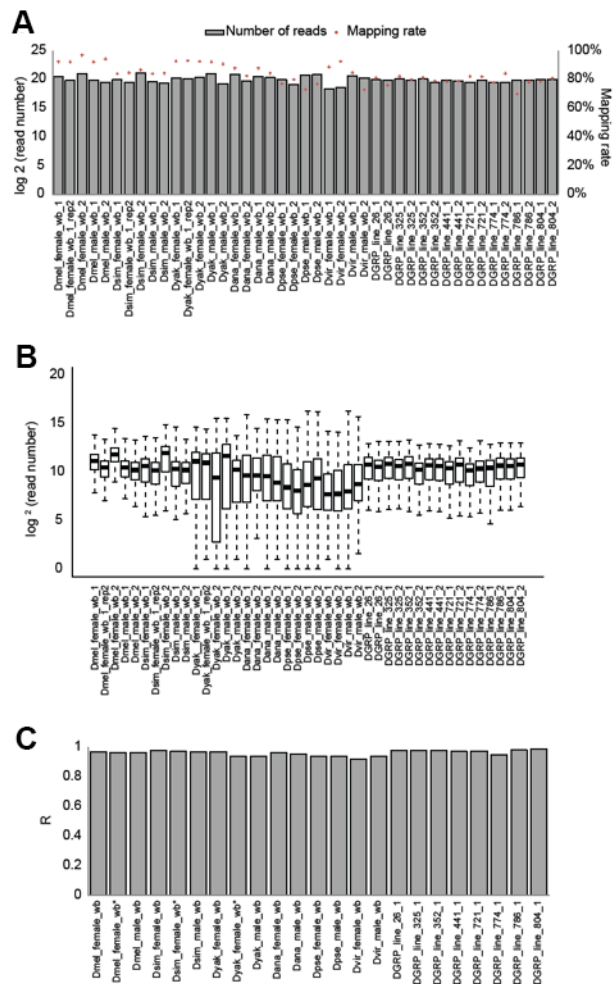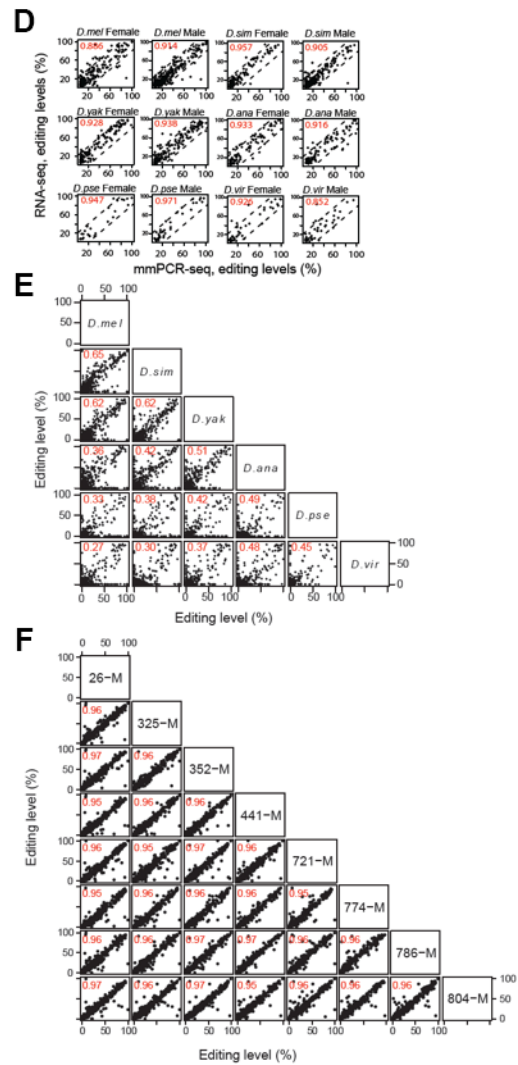

Supplement: S2 Fig — (A) The read number (gray bar) and mapping rate (red dot) of each sample. (B) The coverage of editing sites for each sample. (C) Correlations between editing levels of biological or technical replicates. Sites present in all samples for each species were used for this analysis. All samples are biological replicates except the ones indicated with *. (D) Comparison of editing levels measured by mmPCR-seq and RNA-seq. Both the RNA-seq and mmPCR-seq data are from ~5 day adult whole body samples. A minimum number of 20 and 50 reads is required for sites measured with RNA-seq and mmPCR-seq data, respectively. (E) Pairwise comparison of editing levels between species. The Spearman’s rho values are indicated in red. Female whole body data is shown. (F) Comparison of editing levels measured by mmPCR-seq in eight D.mel strains. Spearman’s rho values are indicated in red. (PDF) [file pgen.1006563.s002.pdf]

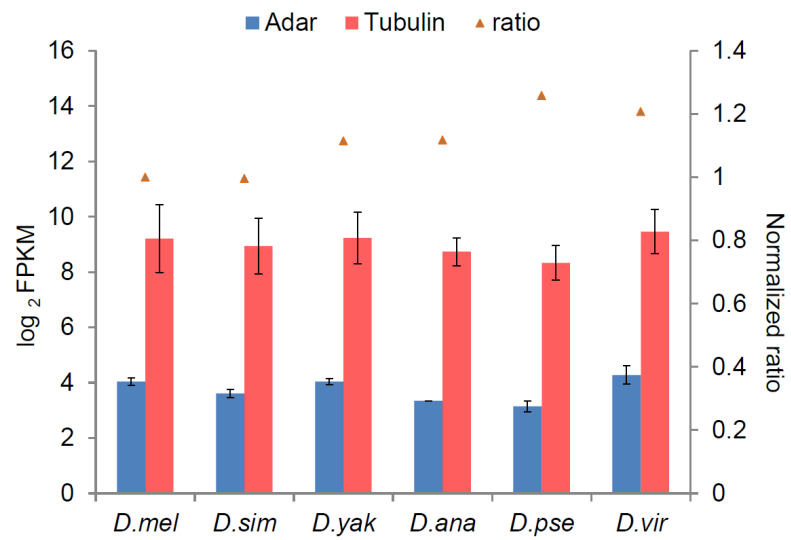

Supplement: S3 Fig — The FPKMs (fragments per kilobase of exon per million fragments mapped) of ADAR and tubulin are shown. The error bars indicate the differences between male and female gene expression levels. The triangles indicate the ADAR expression levels normalized by the tubulin expression levels. (PDF) [file pgen.1006563.s003.pdf]

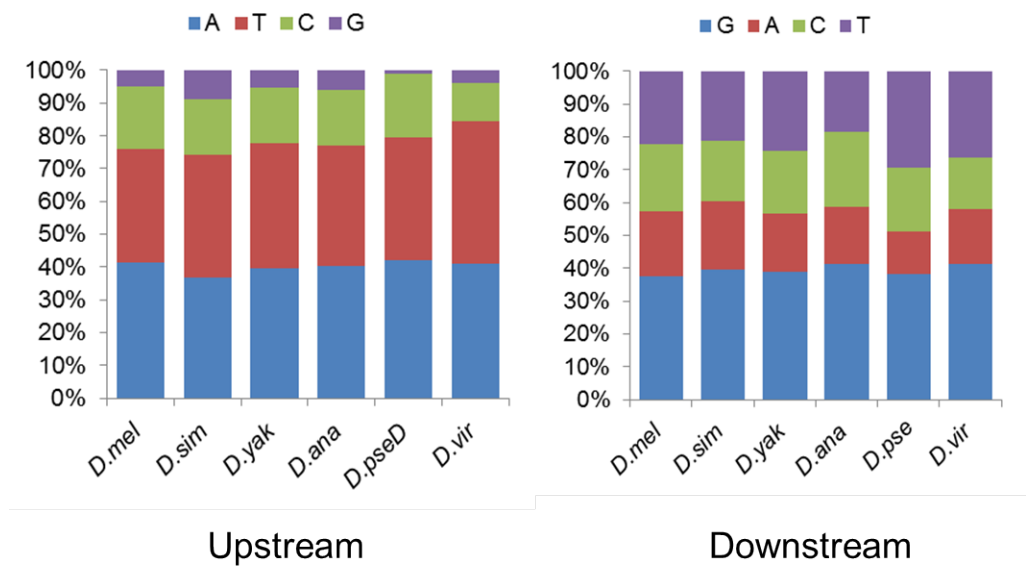

Supplement: S4 Fig — Nucleotide composition in the position immediately upstream (left) and downstream (right) of editing sites in six Drosophila species. (PDF) [file pgen.1006563.s004.pdf]

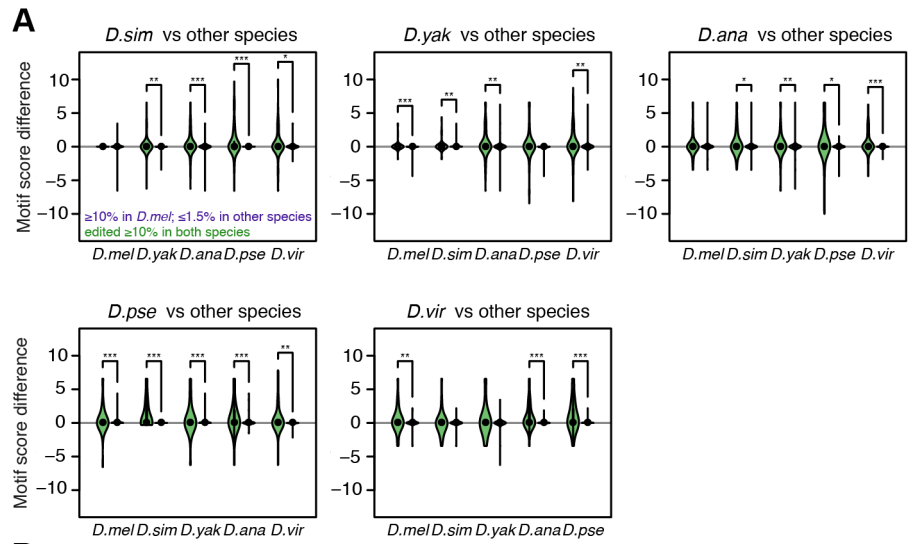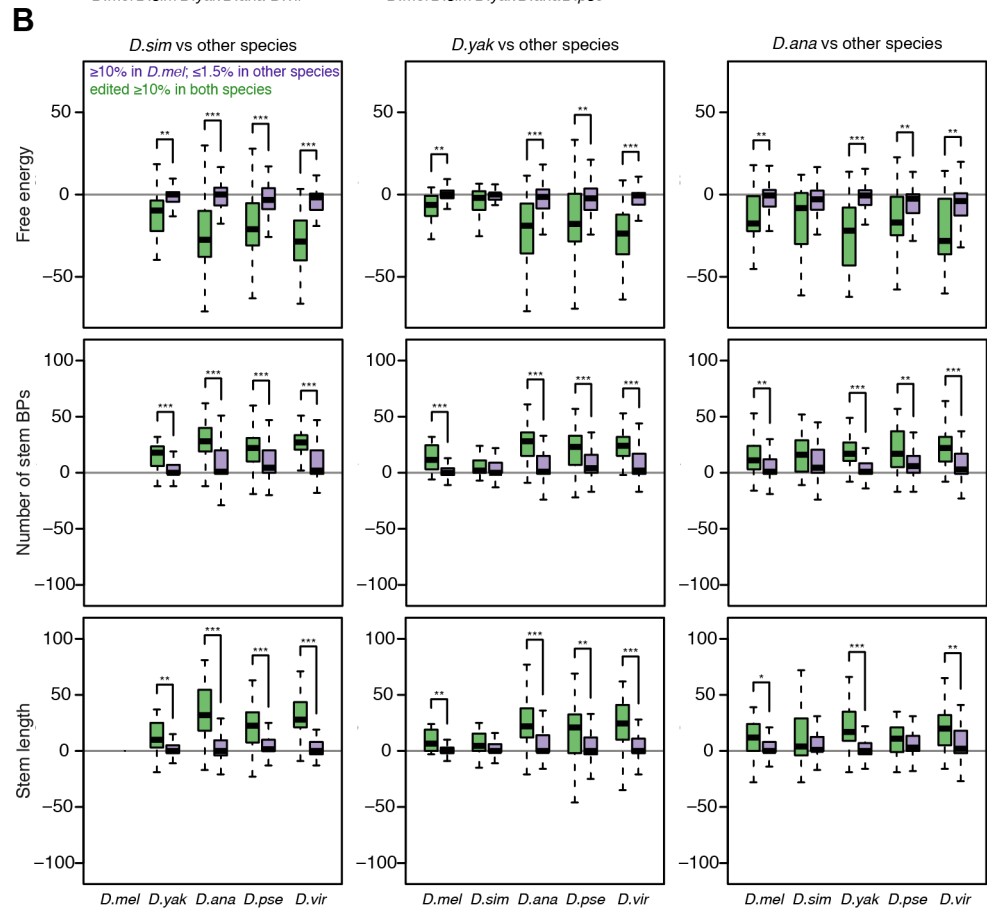

Supplement: S5 Fig — (A) The difference in motif score for editing sites that are edited (≥10%) in one species but not edited (≤1.5%) in the other species under comparison (green), compared to the control in which sites are edited (≥10%) in both species (purple). (B) The difference in free energy, number of paired bases and stem length between sites that are edited (≥10%) in one species but not edited (≤1.5%) in the other species under comparison (green), compared to the control in which sites are edited (≥10%) in both species (purple). The D.mel-D.sim comparisons are not shown because there are fewer than 10 editing sites with ECS predictions that are edited ≥10% in D.sim and not edited in D.mel. D. pse and D. vir are not shown because, for every species pair, fewer than 10 editing sites with ECS predictions are edited ≥10% in them and not edited in the other species under comparison. *: p ≤ 0.05, **: p ≤ 0.01, ***: p ≤ 0.001 (one-tailed Mann-Whitney U test). (PDF) [file pgen.1006563.s005.pdf]

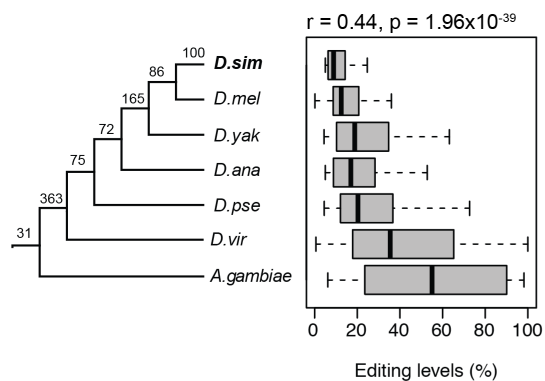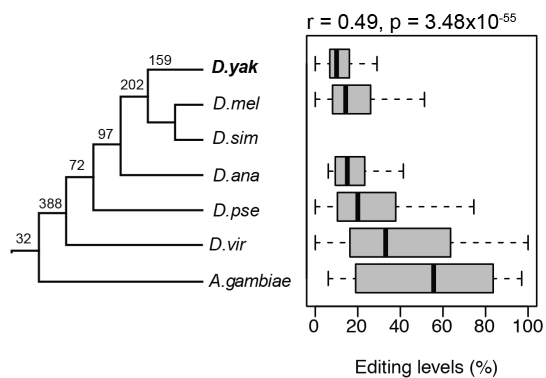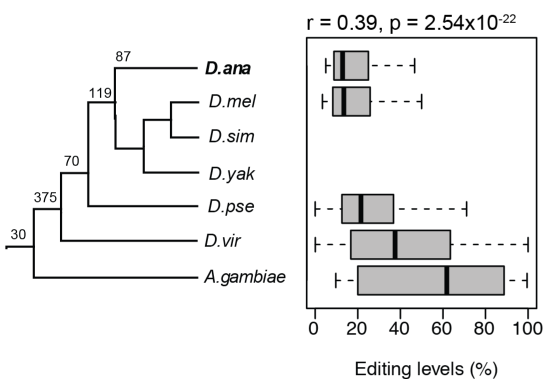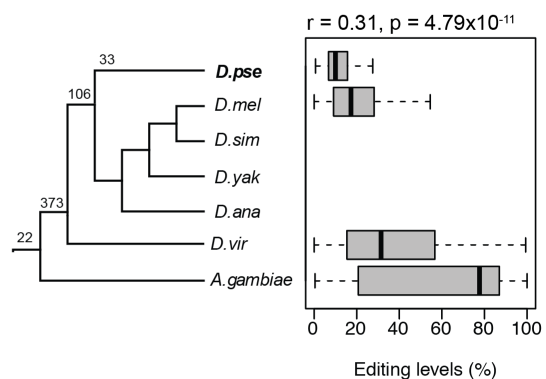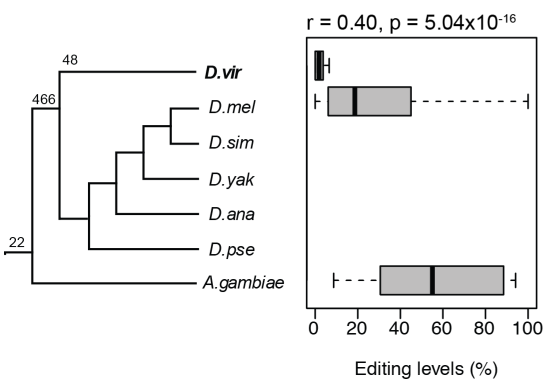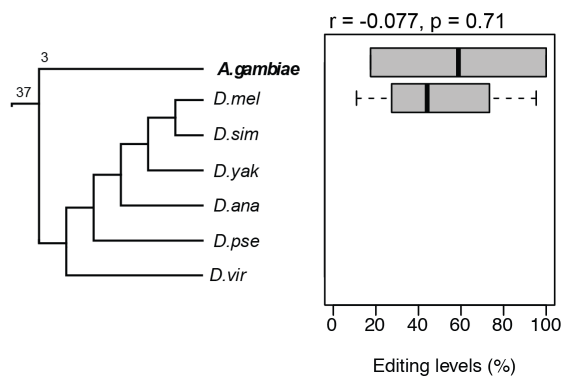

Supplement: S6 Fig — Left of each panel: Depiction of the species and age groups analyzed; numbers above the branches denote the numbers of editing sites in each age group. The anchor species is in bold. Right of each panel: Boxplots of the editing levels in the anchor species for the editing sites in each age group. Spearman’s ρ is indicated. (For the plots using D.mel as the “anchor,” please see Fig 3.) (PDF) [file pgen.1006563.s006.pdf]

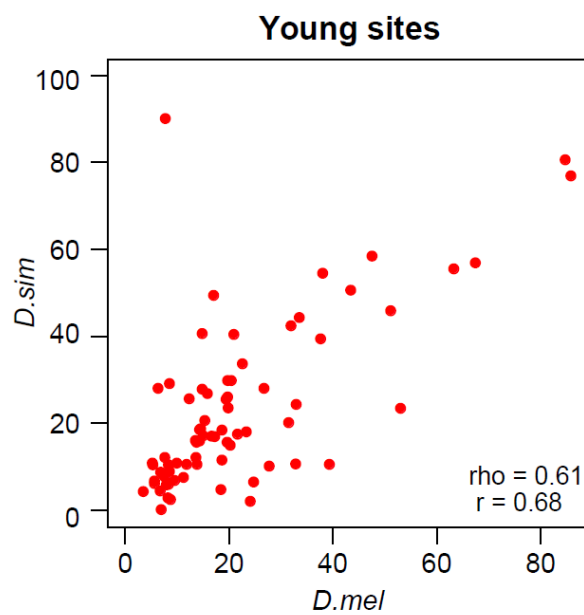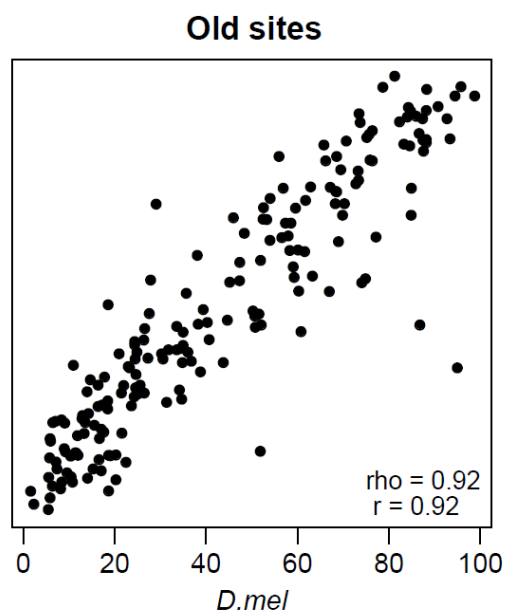

Supplement: S7 Fig — Young sites: shared only in D.mel subgroup (D.mel, D.sim, and D.yak); old sites: sites edited in all Drosophila species analyzed. Both Pearson and Spearman correlation coefficients are shown. (PDF) [file pgen.1006563.s007.pdf]

**A**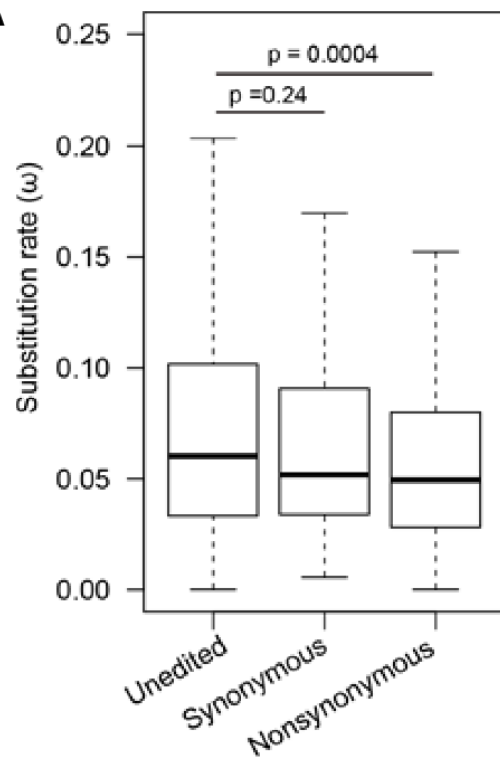**B**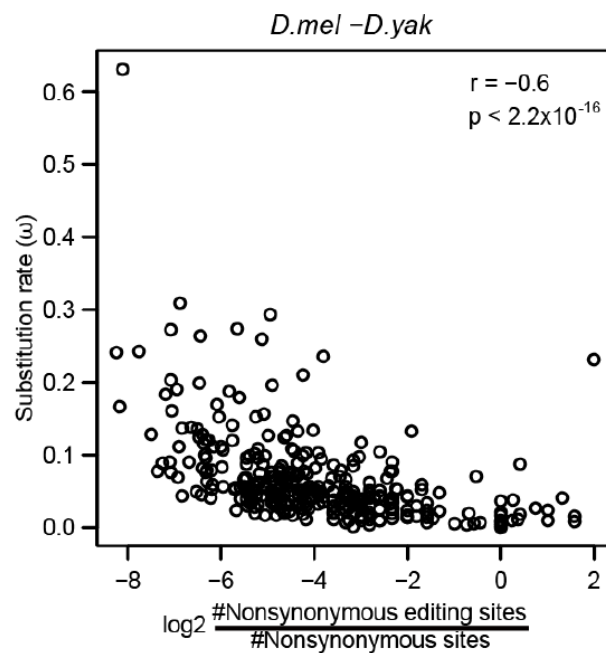**C**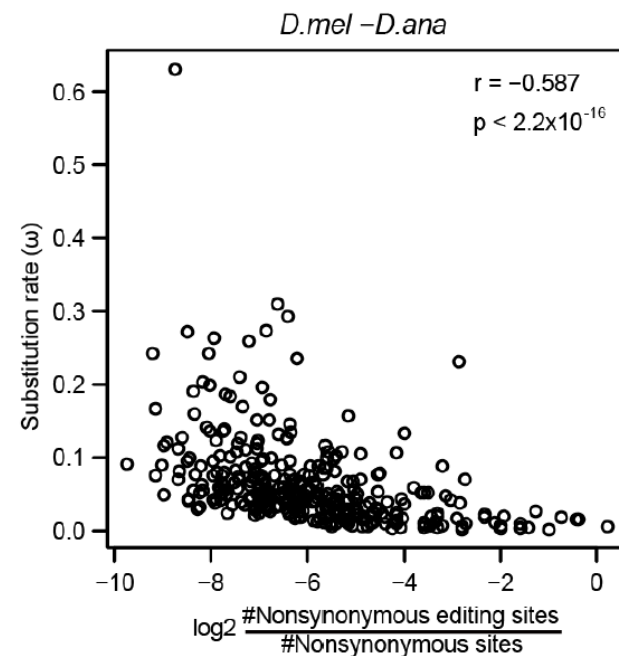**D**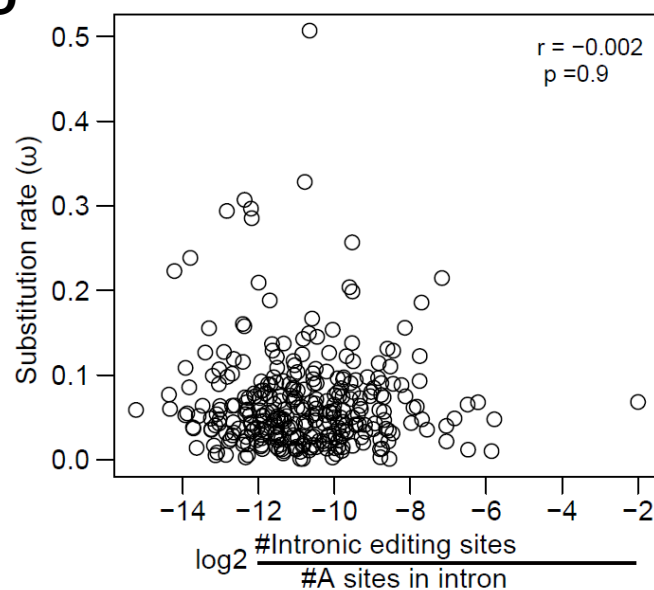**E**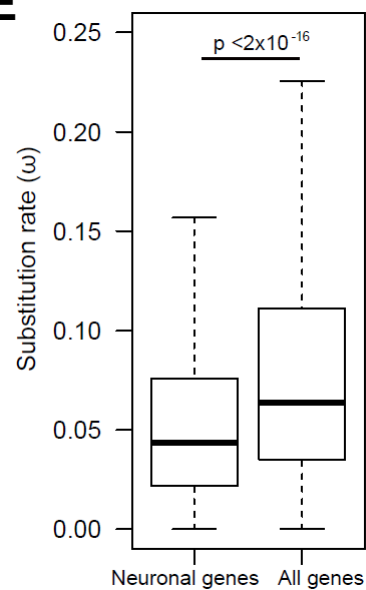

Supplement: S8 Fig — (A) The comparison of evolution rate between genes with nonsynonymous sites, genes with synonymous sites only, and unedited genes. Since editing preferentially occurs in neural tissues, to avoid potential bias, we used genes expressed in heads as a control. (B-C) The relationship between ω of edited genes and the normalized number of nonsynonymous editing sites per gene between D.mel–D.yak (B) or D.mel–D.ana (C). Spearman's ρ is indicated. (D) The relationship between the number of intronic editing sites per gene and omega. The number of intronic editing sites is normalized by the number of adenosines in the intron. (E) The comparison of evolution rate between neuronal genes and all fly protein coding genes. We defined genes with neuron-related GO terms to be neuronal genes. P-values were calculated using the Mann-Whitney U test. (PDF) [file pgen.1006563.s008.pdf]

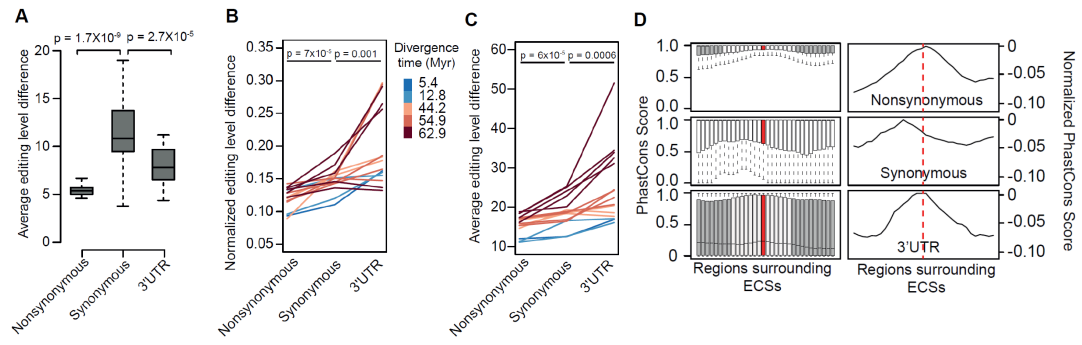

Supplement: S9 Fig — (A) The pairwise comparisons of editing level differences between D.mel strains for nonsynonymous, synonymous, and 3’UTR sites. To minimize the effect of different distributions of editing levels in the three categories, sites edited at ≥20% in one or both strains in the pairwise comparisons were used. P-value was calculated using Paired-sample Wilcoxon test. (B-C) The pair-wise comparisons of normalized (B) or unnormalized (C) average editing level difference across species. Male whole body data was shown. All sites with at least 20 reads are included. Sites edited at ≥20% in one or both strains in the pairwise comparisons were used. P-value was calculated using the Paired-sample Wilcoxon test. (D) Conservation of DNA sequences surrounding ECSs. Left: the PhastCons score distributions for the 60 bp regions flanking the corresponding sites in ECSs that are paired with the editing sites. The results were plotted separately for nonsynonymous, synonymous and 3’UTR editing sites using a 30 bp window size. We only analyzed D.mel proximal ECSs, in order to avoid potential bias of using evolutionarily conserved intronic ECSs. Since ECSs located in different genic regions likely have different evolution rates, for nonsynonymous and synonymous editing sites, we only examined ECSs located in CDS regions; for 3’UTR editing sites, we only examined ECSs located in 3’UTR regions. Regions that are significantly different (Kolmogorov-Smirnov Tests, fdr corrected p ≤ 0.01 and D ≥ 0.05) from the site paired with editing site (centered, colored red) are colored gray. Right: normalized conservation score, i.e. the D statistic of the flanking regions of edited loci relative to the region with the highest conservation score. (PDF) [file pgen.1006563.s009.pdf]

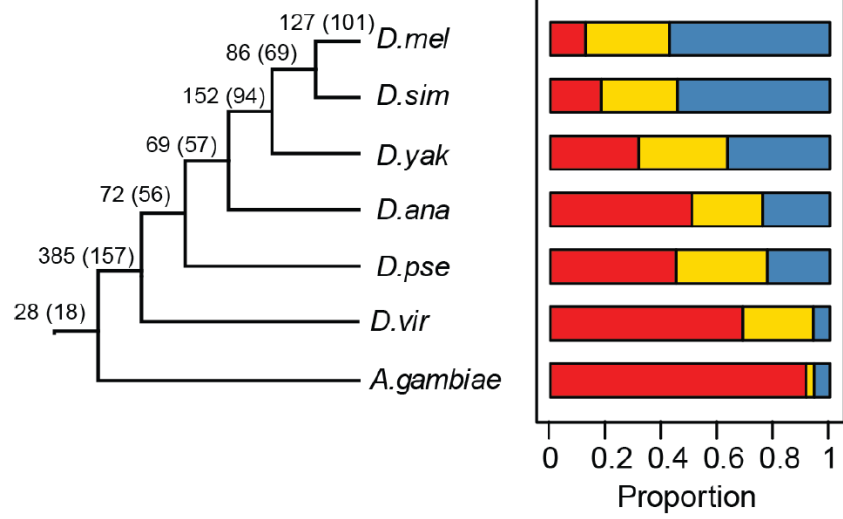

Supplement: S10 Fig — Left: Depiction of the species and age groups analyzed as in Fig 3A. Right: The distribution of highly (red), moderately constrained (yellow), or unconstrained (blue) sites in each age group. (PDF) [file pgen.1006563.s010.pdf]

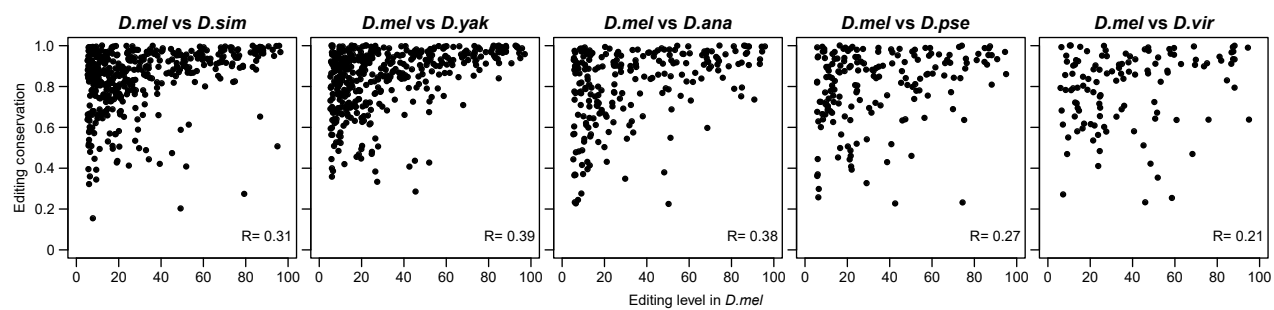

Supplement: S11 Fig — Editing conservation is defined as 1 − Absolute value (editing level, D.mel–editing level, the other species) / (editing level, D.mel + editing level, the other species). Spearman's ρ is indicated. (PDF) [file pgen.1006563.s011.pdf]
